# Awareness of Urinary Stone Risk Factors Among the Adult Population of Jazan, Saudi Arabia: A Cross-Sectional Study

**DOI:** 10.7759/cureus.49115

**Published:** 2023-11-20

**Authors:** Essa Adawi, Naif K Mahzara, Rafa Hadaddi, Seham Ageeli, Tahani Altubayqi, Esaam Moafa, Sultan Althurwi, Alyaj A Hakami, Reem Qahtani, Abdulaziz M Kariri, Sumayyah Jafar, Taif Kobal, Rafa Alqaari

**Affiliations:** 1 Urology, Jazan University, Jazan, SAU; 2 Medicine, Jazan University, Jazan, SAU

**Keywords:** jazan, nutritional factors, risk factors, urinary stone, urolithiasis

## Abstract

Background: Urolithiasis, a common clinical condition, has seen a global increase in prevalence in recent years. Urinary stones are common in hot climate areas like Saudi Arabia. This study aimed to assess the prevalence and awareness of risk factors for urinary stones in Jazan, Saudi Arabia.

Methods: A cross-sectional study was conducted among 1000 Jazan adults between January and June 2022. A questionnaire was used to collect data on sociodemographics, urinary stone diagnosis, and awareness of risk factors.

Results: The overall prevalence of diagnosed urinary stones was 140 (14.0%). The prevalence was higher among patients who were older, married, had higher education, and were employees (all P<0.001). Participants who believed that hot weather (p = 0.012), sleeve gastrectomy (p = 0.049), and Saxenda injections (p = 0.000) increased the risk of stones had a higher prevalence. No association was found between stones and other sociodemographic factors or dietary habits. The main sources of knowledge were the internet (426, 42.6%) and education (155, 15.5%).

Conclusions: The prevalence of urinary stones in Jazan is considerable (14.0%). Certain sociodemographic factors and beliefs about risk factors were associated with higher prevalence. Improving public awareness about the prevention and risk factors for urinary stones is crucial to controlling this health problem in high-risk communities.

## Introduction

Nephrolithiasis or kidney stones are a common problem that often progresses to other chronic complications, which can cause distress [1]. Mineral deposits in kidney stones can be free or connected to the renal papillae in the renal calyces and pelvis. When urine reaches the supersaturation point of a mineral, it begins to form and contains both crystalline and organic components [2]. Recently, there has been a significant global increase in the prevalence of kidney stones. Regarding the prevalence in Saudi Arabia, a previous study on Hail has shown a high prevalence [3]. Another study has shown that the prevalence among males at 15.7% was higher than 9.4% among females. However, the prevalence was approximately the same among Saudis and non-Saudis [4].

The etiology of nephrolithiasis involves several factors and is closely connected to disregarding a healthy lifestyle [5]. Low fluid intake and high-fat diets are considered the most critical factors that play an essential role in stone formation of stones formation [6]. Therefore, patients with recurrent kidney stones are advised to follow a diet that limits their sodium and protein intake [7]. In addition, other nutritional factors include energy drinks, black tea, chocolate consumption, and low vegetable intake [6]. The risk of kidney stones has been linked to higher fructose intake. A low-calorie diet can reduce the prevalence of stones [7]. Obesity and being overweight are closely related to an increased risk of developing stones in both sexes [8]. The effect of a particular diet common in the Gulf region and the Middle East is believed to cause the high incidence and prevalence of kidney stone disorders in the Kingdom of Saudi Arabia. This diet is characterized by low calcium intake and excessive animal protein and oxalate intake, leading to hyperoxaluria and an increased risk of nephrolithiasis. In addition, urine quantities were lower than normal due to the hot and dry environment, raising the possibility of stone formation [9,10].

Furthermore, other dietary factors such as low fiber intake, high starch intake, and high purine-rich foods can increase risk of kidney stones. A diet low in fiber can lead to constipation and reduced fluid intake, while diets high in refined carbohydrates and starch increase acidity in urine, promoting stone formation. Purine-rich foods increase uric acid levels in urine, which can crystallize into stones [9].

Meanwhile, non-nutritional factors contribute to the formation of stones, such as older age, male sex, family history, and high-temperature exposure, making the population living in the hot regions of the kingdom more likely to develop stones [3]. Most stone formers react differently to changes in food patterns and environmental conditions. As a result, medical care for kidney stones must include modifying dietary habits and avoiding unfavorable environmental conditions [10]. Public awareness about kidney stones can lead to healthier lifestyle choices, potentially preventing stone formation and improving overall health. However, research assessing the level of public awareness about kidney stone risk factors is limited. Therefore, this study aims to evaluate the level of public awareness regarding the risk factors for kidney stones, with the hope that improved awareness will contribute to future prevention efforts.

## Materials and methods

Study design and study settings 

A descriptive cross-sectional study was conducted between January and June 2022 among the adult Jazan population. Jazan is one of the 13 major regions in Saudi Arabia. According to the 2016 census, the population of Jazan is 1.568 million people.

Study population

All members of the Jazan population who met the inclusion criteria were involved in the study.

Inclusion and exclusion criteria

Individuals who resided in the Jazan region, aged 18 and older, and were willing to participate were included. Individuals who refused to participate, those under 18, or those who resided outside the Jazan region were excluded.

Sampling method

A multi-stage cluster sampling method was used to select participants across the Jazan region. In cluster sampling, the population is divided into clusters (groups) first, and then samples are randomly selected from each cluster. To begin, the Jazan region was divided into several clusters based on administrative divisions or geographic areas. The number and boundaries of the clusters were determined to allow a representative sampling of the population. Next, a random sample of these clusters was selected to participate in the study. The probability of selection was proportional to the population size of each cluster to ensure proper representation. Within the selected clusters, participants were conveniently recruited by data collectors using online questionnaires. However, the clusters themselves were randomly determined initially. This multi-stage cluster sampling approach was utilized due to the large target population size dispersed across the entire Jazan region. Cluster sampling allowed for more feasible recruitment of participants compared to simple random sampling in this cross-sectional health study.

Sample size

The sample size for this study was determined using a statistical formula for probability sampling. Specifically, the sample size was calculated based on the desired confidence level, anticipated population proportion, and acceptable margin of error.

The formula used was: n = z^2*(1-α) * P*(1-P) / d^2

Where: n is the required sample size, z is the z-score corresponding to the desired confidence level (1.96 for 95% confidence), α is the significance level (typically 0.05), P is the anticipated population proportion (0.50 was used to obtain the maximum sample size), and d is the acceptable margin of error (0.05 was used).

Applying this formula, the minimal sample size was 385, but this study collected 1000 participants. However, the sample size was further adjusted to account for an expected non-response rate. Assuming a 10% non-response rate, the final target sample size was determined to be 425 participants. This approach ensured that the sample size was large enough to provide statistically meaningful results within an acceptable margin of error while accounting for the likelihood of some non-responses. The use of the probability sampling formula allowed the calculation of a representative sample size from the broader Jazan population.

Data collection tools and processes

Data were collected by multiple data collectors using a self-administered online questionnaire. To reduce potential inter-observer bias, all data collectors underwent training on the standardized administration of the online questionnaire and procedures for interacting with participants. The online questionnaire was designed and hosted using an online survey platform. Links to access the online questionnaire were shared with potential participants by the data collectors through email and text messaging. Additionally, the survey link was promoted through social media platforms and online groups focused on the Jazan region to reach a wider audience. Multiple trained data collectors were responsible for the recruitment and distribution of the electronic survey link to eligible participants in their assigned area clusters. The data collectors sent invitation messages containing information about the study and a personalized link for the online questionnaire. Reminder messages were sent on a weekly basis to contact individuals who had not yet completed the survey. The online questionnaire was designed based on an extensive literature review of similar studies [1,3] and in consultation with an expert in the field to ensure appropriate data collection. It consisted of close-ended questions organized into three sections: sociodemographics, urinary stone awareness, and urinary stone risk factors. Participants were able to complete the anonymous online questionnaire at their convenience within the data collection period.

Pilot study

A pilot study was conducted within the target population to assess the clarity and understandability of the questionnaire language. The pilot study data were analyzed to determine the internal consistency and reliability of the questionnaire scales using Cronbach's alpha. Values above 0.7 would indicate acceptable reliability. Any negative values or problematic questions would be revised to improve the internal reliability of the scales. The finalized questionnaire underwent reliability testing again in the main study population. Only the main study data was included in the final results and analysis. The validated Arabic version of the questionnaire was administered to all participants in the main study following the pilot.

Data entry and statistical analysis

Data management and analysis were performed on the SPSS version 26 (IBM Corp., Armonk, NY, USA). Frequencies and percentages were used for qualitative data; for quantitative data, means and standard deviations were used. The Chi-square and Fisher's exact tests were used to compare categorical data, while the T-test was used for continuous variables. A P-value of 0.05 indicated a statistically significant difference between the variables. Tables and figures were used to express the results.

Ethical considerations

Ethical approval was obtained from the Jazan University in Saudi Arabia, REC-44/08/588. Additionally, each participant received an electronic copy of a letter of information posted online to give their electronic permission. It was included as a preliminary cover page before starting their online questionnaire. Respect for the dignity of the research participants was prioritized. All participants had the right to withdraw from the study at any time. All participant data was kept in a secure location with high confidentiality.

## Results

The questionnaire was completed by 1000 agreed participants, 466 (46.6%) male and 534 (53.4%) female. Most participants were Saudi (969, 96.9%), and the remaining 31 (3.1%) were non-Saudi. Regarding marital status, 535 (53.5%) were single, 433 (43.3%) were married, 20 (2.0%) were divorced, and 12 (1.2%) were widowed. Most participants had a university education (586, 58.6%), 197 (19.7%) had a secondary education, and 186 (18.6%) were graduates. Employee (415, 41.5%) was the most common occupation, followed by student (397, 39.7%). The mean age of the participants was 30.82 ± 10.47 years, with a mean height of 162.49 ± 10.23 cm and a mean weight of 68.90 ± 19.77 kg (Table 1).

**Table 1:** Sociodemographic characteristics of 1000 participants N: Number, (%): percentage, SD: standard deviation, cm: centimeter, kg: kilogram

Sociodemographic		N	%
Gender	Male	466	46.6
	Female	534	53.4
Nationality	Saudi	969	96.9
	Non-Saudi	31	3.1
Marital status	Single	535	53.5
	Married	433	43.3
	Divorced	20	2.0
	Widower	12	1.2
Educational level	University	586	58.6
	Secondary	197	19.7
	Graduate	186	18.6
	Middle	18	1.8
	Primary	12	1.2
	Uneducated	1	0.1
Occupation	Student	397	39.7
	Employee	415	41.5
	Unemployed	153	15.3
	Retired	35	3.5
Sociodemographic		Mean	SD
Age by year		30.82	± 10.47
Height by cm		162.49	± 10.23
Weight by kg		68.90	± 19.77

**Figure 1:**
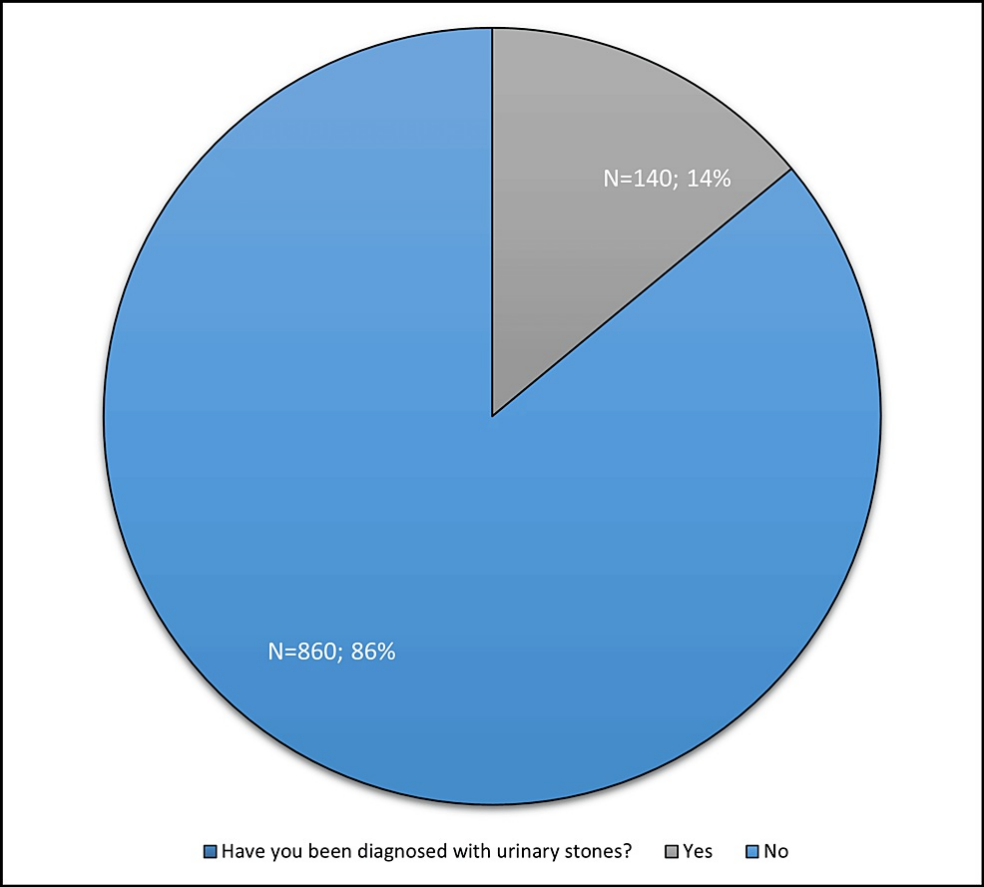
Prevalence of diagnosed urinary stones among 1000 respondents N: Number, (%): percentage

Of the total number of respondents, 140 (14.0%) reported having urinary stones, while 860 (86.0%) reported not being diagnosed with urinary stones diagnosis (Figure 1).

According to the results, the prevalence of diagnosed urinary stones among respondents was based on various sociodemographic characteristics. Respondents who have been diagnosed with urinary stones have a mean age of 36.03 ± 11.76 years compared to those who have not been diagnosed, who have a mean age of 29.97 ± 10.33 years. Married respondents have a higher prevalence of diagnosed urinary stones (8.3%, n = 83) than single (5.0%, n = 50), divorced (0.4%, n = 4), or widowed (0.3%, n = 3) respondents. Those with university education have a higher prevalence (6.8%, n = 68) than those with secondary (3.4%, n = 34), graduate (3.2%, n = 32), middle school (0.1%, n = 1), primary (0.4%, n = 4), or no education (0.1%, n = 1). Employed respondents have a higher prevalence (8.1%, n = 81) than students (2.4%, n = 24), unemployed (2.2%, n = 22), or retired (1.3%, n = 13) respondents. There is an association between being diagnosed with urinary stones and age (p = 0.000), marital status (p = 0.000), education (p = 0.004), and occupation (p = 0.000). This difference is statistically significant (p < 0.05). There is no significant difference in the prevalence of diagnosed urinary stones between males (7.2%, n = 72) and females (6.8%, n = 68), Saudi nationals (13.4%, n = 134), and non-Saudi nationals (0.6%, n = 6). There is no association between the diagnosis of urinary stones and gender (p = 0.235), nationality (p = 0.426), height (p = 0.453), or weight (p = 0.633) (Table 2).

**Table 2:** Association between diagnosed urinary stones among respondents and sociodemographic characteristics. N: Number, (%): percentage, SD: standard deviation P: Pearson's chi-squared test X2, $: exact probability test, * P < 0.05 (significant)

Sociodemographic		Have you been diagnosed with urinary stones?		P-Value
		Yes N (%)	No N (%)	
Age by year; Mean ± SD		36.03 ± 11.76	29.97 ± 10.33	0.000*
Height by cm; Mean ± SD		163.10 ± 10.34	162.40 ± 10.21	0.453
Weight by kg; Mean ± SD		68.16 ± 17.54	69.02 ± 20.12	0.633
Gender	Male	72 (7.2%)	394 (39.4%)	0.235^$^
	Female	68 (6.8%)	466 (46.6%)	
Nationality	Saudi	134 (13.4%)	835 (83.5%)	0.426^$^
	Non-Saudi	6 (0.6%)	25 (2.5%)	
Marital status	Single	50 (5.0%)	485 (48.5%)	0.000*
	Married	83 (8.3%)	350 (35.0%)	
	Divorced	4 (0.4%)	16 (1.6%)	
	Widower	3 (0.3%)	9 (0.9%)	
Educational level	University	68 (6.8%)	518 (51.8%)	0.004*
	Secondary	34 (3.4%)	163 (16.3%)	
	Graduate	32 (3.2%)	154 (15.4%)	
	Middle	1 (0.1%)	17 (1.7%)	
	Primary	4 (0.4%)	8 (0.8%)	
	Uneducated	1 (0.1%)	0 (0.0%)	
Occupation	Student	24 (2.4%)	373 (37.3%)	0.000*
	Employee	81 (8.1%)	334 (33.4%)	
	Unemployed	22 (2.2%)	131 (13.1%)	
	Retired	13 (1.3%)	22 (2.2%)	

Respondents who believe that working in hot weather increases the risk of urinary stones have a higher prevalence of diagnosed urinary stones (5.9%, n = 59), compared to those who do not believe it (3.9%, n = 39) or are unsure (4.2%, n = 42). Respondents who believe that sleeve gastrectomy increases the risk of urinary stones have a higher prevalence of diagnosed urinary stones (4.7%, n = 47) compared to those who do not believe it (2.2%, n = 22) or are unsure (7.1%, n = 71). Respondents who believe that injections of Saxenda increase the risk of urinary stones have a higher prevalence of diagnosed urinary stones (5.7%, n = 57) compared to those who do not believe it (0.7%, n = 7) or are unsure (7.6%, n = 76). All these risk factors are statistically significant with a diagnosis of urinary stones (p < 0.05) (Table 3).

**Table 3:** Association between diagnosed urinary stones among respondents and risk factors. N: Number, (%): percentage P: Pearson's chi-squared test X2, * P < 0.05 (significant)

Risk factors		Have you been diagnosed with urinary stones?		P-Value
		Yes N (%)	No N (%)	
Drinking water prevents urinary stones.	Yes	120 (12.0%)	737 (73.7%)	0.580
	No	8 (0.8%)	35 (3.5%)	
	I don't know	12 (1.2%)	88 (8.8%)	
Working in hot weather increases urinary stones.	Yes	59 (5.9%)	259 (25.9%)	0.012*
	No	39 (3.9%)	254 (25.4%)	
	I don't know	42 (4.2%)	347 (34.7%)	
Fat food increases urinary stones.	Yes	85 (8.5%)	533 (53.3%)	0.887
	No	19 (1.9%)	122 (12.2%)	
	I don't know	36 (3.6%)	205 (20.5%)	
Salt reduces the appearance of urinary stones.	Yes	91 (9.1%)	567 (56.7%)	0.627
	No	15 (1.5%)	110 (11.0%)	
	I don't know	34 (3.4%)	183 (18.3%)	
Calcium intake reduces the occurrence of urinary stones.	Yes	55 (5.5%)	266 (26.6%)	0.130
	No	36 (3.6%)	235 (23.5%)	
	I don't know	49 (4.9%)	359 (35.9%)	
Reducing soft drinks reduces the incidence of urinary stones.	Yes	104 (10.4%)	601 (60.1%)	0.231
	No	16 (1.6%)	84 (8.4%)	
	I don't know	20 (2.0%)	175 (17.5%)	
Reducing coffee consumption reduces urinary stones.	Yes	87 (8.7%)	440 (44.0%)	0.052
	No	23 (2.3%)	173 (17.3%)	
	I don't know	30 (3.0%)	247 (24.7%)	
Sleeve gastrectomy increases urinary stones	Yes	47 (4.7%)	207 (20.7%)	0.049*
	No	22 (2.2%)	172 (17.2%)	
	I don't know	71 (7.1%)	481 (48.1%)	
Urine retention increases urinary stones.	Yes	107 (10.7%)	613 (61.3%)	0.256
	No	13 (1.3%)	73 (7.3%)	
	I don't know	20 (2.0%)	174 (17.4%)	
Saxenda injections increase the risk of urinary stones.	Yes	57 (5.7%)	164 (16.4%)	0.000*
	No	7 (0.7%)	78 (7.8%)	
	I don't know	76 (7.6%)	618 (61.8%)	

Respondents who believe that calcium intake reduces the risk of kidney stones have a lower prevalence of diagnosed kidney stones (5.5%, n = 55) compared to those who do not believe it (3.6%, n = 36) or are unsure (4.9%, n = 49). However, this difference is not statistically significant. Furthermore, respondents who believe that reducing coffee consumption reduces the risk of kidney stones have a slightly lower prevalence of diagnosed kidney stones (8.7%, n = 87) compared to those who do not believe it (2.3%, n = 23) or are unsure (3.0%, n = 30). However, this difference is not statistically significant (Table 3). There is no association between being diagnosed with urinary stones and drinking water (p = 0.580), fat food consumption (p = 0.887), salt consumption (p = 0.627), calcium intake (p = 0.130), soft drinks (p = 0.231), coffee consumption (p = 0.052), or urinary retention (p = 0.256) (Table 3).

**Figure 2:**
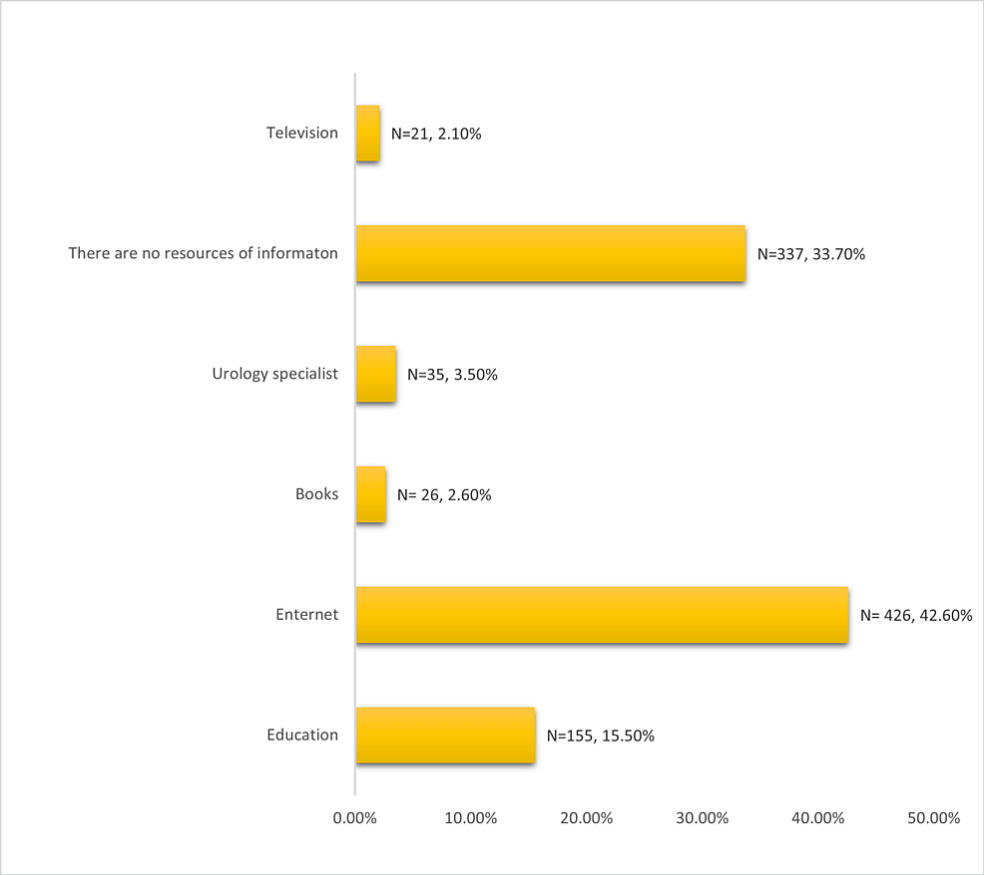
Sources of information about urinary stones of respondents. N: Number, (%): percentage

Figure 2 shows the sources of information about urinary stones among the respondents. Most of the respondents (426, 42.6%) reported using the internet, 337 (33.7%) of the respondents reported not having any resources for information on urinary stones, 155 (15.5%) of the respondents reported education, a small proportion of the respondents (35, 3.5%) reported consulting a urologist, only 26 (2.6%) of the respondents reported using books, and a tiny proportion of the respondents (21, 2.1%) reported television as a source of information on urinary stones.

## Discussion

The study results provide a snapshot of the prevalence and risk factors of urinary stones among the participants and their sources of information on the condition. Most of the participants were Saudi, educated at the university, employed, or students.

The study found that the overall prevalence of diagnosed urinary stones among the respondents was 140 (14.0%). This result is consistent with previous studies reporting that the prevalence of urolithiasis in Saudi Arabia is 13.7% in the Hail region [3], 11.2% in Jeddah [4], and 19.1% in Riyadh [11]. At the national level, the prevalence is 9.1% in Saudi Arabia [12]; another study reported 20.1% in Saudi Arabia [13]. However, the worldwide prevalence rate ranges from 4% to 20% [14].

In our study, a higher prevalence of urinary stones was associated with increased age, like previous studies' findings. According to research by Scales et al., kidney stones became more common as people grew up, reaching a high in the age range of 60-69 [15]. Similarly, a systematic review found that age is a significant risk factor for urinary stones, and middle-aged people have the highest incidence rates [16]. This can be attributed to age-related changes, such as decreased urine volume and increased solute concentration, which can promote stone formation [17,18]. Hormonal changes caused by aging, such as lower estrogen levels in postmenopausal women, can promote bone resorption and release more calcium into the circulation and urine. This may make calcium stones more likely to develop [19]. Furthermore, comorbid diseases, including obesity, diabetes, and hypertension, are more prevalent in older people and are associated with an increased risk of developing urinary stones [20].

A current study reveals that marital status, educational level, and occupation were also significantly associated with the diagnosis of urinary stones. Respondents who were married, had a university education, and were employed had a higher prevalence of diagnosed urinary stones compared to other groups. It is plausible that individuals who are married or have higher educational levels are typically older compared to those who are single or have a lower education. Older age has been established as a significant risk factor for urinary stones in several studies, including ours. Therefore, the observed associations might be attributed, at least in part, to the age differences across these groups rather than the direct effect of marital status, educational level, or occupation on the prevalence of urinary stones. Our findings are consistent with previous research, which has shown that urinary stones are more common among older people, married individuals, and those with higher levels of education and certain occupations [21]. For instance, studies have reported a higher risk of urinary stones among older individuals [16], those with higher education levels [22], and specific occupations [23]. This contrasts with other studies that found no association between socioeconomic status and prevalence of urolithiasis [24]. However, it should be noted that the study did not find a significant association between gender, nationality, height, or weight and the prevalence of urinary stones. Other studies have not reported any significant association between these factors and urinary stone diagnosis [21].

Our results revealed that in terms of stone risk factors, the belief that working in hot weather, sleeve gastrectomy, and Saxenda injections increase the risk of urinary stones was associated with a higher prevalence of diagnosed stones. This suggests that respondents may be aware of possible risk factors for urinary stones. Furthermore, this perception reflects the awareness that dehydration, rapid weight loss, and obesity can contribute to stone formation [25]. However, in our study, other well-established risk factors, such as low fluid intake, high salt or protein intake, and inadequate calcium intake, were not significantly associated with stone diagnosis. Previous research reported the need for public education about stone prevention, proper hydration, diet, and lifestyle modification [26]. In addition, no significant associations were found between urinary stone diagnosis and various dietary habits or practices, such as drinking water, eating fat, salt, calcium intake, soft drink consumption, coffee consumption, or urinary retention. This may suggest that these factors are not strong predictors of the development of urinary stones in this population. However, more research using longitudinal designs is needed to understand better these factors' role in developing urinary stones. The study provides information on the prevalence and perception of urinary stone disease in Saudi Arabia. The findings suggest that the prevalence of urolithiasis is substantial and that more education and awareness are needed about stone prevention. Targeted strategies for groups at high risk of developing stones, such as elderly and obese individuals, should be implemented to reduce the overall burden of the disease.

Most respondents reported using the Internet as their main source of information on urinary stones. This finding highlights the growing importance of the Internet as a source of health information and the potential for misinformation, as not all online resources provide accurate and evidence-based information [27]. This underscores the need for healthcare professionals and organizations to easily access correct and reliable information about urinary stones through online platforms. A significant proportion of the respondents also reported that they did not have a source of information on urinary stones, highlighting the need for greater public awareness and education on this health issue.

Adequate fluid intake is widely recognized as a key preventive measure for kidney stones, regardless of composition or risk factors [28]. Public education on maintaining optimal hydration could significantly reduce the burden of urinary stones [29]. Moreover, hot climates lead to increased sweat losses, decreased urinary volume, and concentrated urine, all of which promote crystallization [24]. Rising temperatures from climate change may increase the risk of kidney stones in vulnerable populations [30]. Various modern technologies can aid health literacy efforts in this population. Mobile health apps demonstrate high patient engagement and improvement in knowledge regarding nephrolithiasis [31]. Social media campaigns also effectively improve awareness of kidney disease risk factors and prevention behaviors [32]. Gamified hydration apps are an especially innovative approach to increasing water intake through tailored goals, reminders, and incentives [32,33]. It is recommended that healthcare professionals collaborate with public health agencies and experts to develop culturally appropriate educational campaigns on stone prevention. Multidisciplinary collaboration between various stakeholders is key for successful public health campaigns on urolithiasis in this community.

This study provides valuable information on the prevalence, sociodemographic characteristics, and sources of information related to urinary stones in a specific population. The findings highlight the importance of reliable and accessible health information and the need for further research to understand better the role of various risk factors in developing urinary stones. The findings underscore the importance of public education and awareness campaigns to help people better understand the risk factors associated with urinary stones and to take appropriate preventive measures.

Study limitations

This study has various limitations that should be highlighted. The study used a cross-sectional design, which can only show associations but not prove causality. The study relied on self-reported data collected through a questionnaire, which could lead to recall bias. Due to the survey conducted in the Jazan region, our findings cannot be generalized to the entire kingdom of Saudi Arabia.

## Conclusions

The study found that the overall prevalence of diagnosed urinary stones among the respondents was 14.0%. The prevalence was significantly higher among older participants, women, married individuals, those with higher education, and employees. Participants who believed that certain risk factors increase the risk of urinary stones, such as hot weather, sleeve gastrectomy, and Saxenda injections, had a higher prevalence of stones. However, no significant association was found between urinary stones and other sociodemographic factors or dietary habits. Most respondents relied on the internet as their primary source of information about urinary stones, followed by education, urologists, books, and television. Effective awareness campaigns on risk factors and prevention measures can help reduce the prevalence of urinary stones in the community. Targeted education and lifestyle modifications can benefit high-risk groups. The results provide valuable information on the factors influencing urinary stones in the Saudi population, which can guide prevention and management strategies.
